# ImpENSA eHealthy Conversation Skills training for healthcare professionals aimed at improving micronutrient status during the first 1000 days in South Africa

**DOI:** 10.1371/journal.pgph.0003833

**Published:** 2024-12-04

**Authors:** Bernadeta Patro-Golab, Sunhea Choi, Jan Lukasik, Corinna Walsh, Maciej Kolodziej, Lize Havemann-Nel, Estelle Venter, Kerry Sexton, Selma Omer, Liz Goddard, Keith M. Godfrey, Wendy Lawrence, Berthold Koletzko, ImpENSA Study Group

**Affiliations:** 1 Department of Paediatrics, Medical University of Warsaw, Warsaw, Poland; 2 Department of Paediatrics, Dr. von Hauner Children’s Hospital, LMU-Ludwig Maximilians Universität Munich, Munich, Germany; 3 German Center for Child and Adolescent Health, Munich, Germany; 4 Faculty of Medicine, University of Southampton, Southampton, United Kingdom; 5 Department of Nutrition and Dietetics, University of the Free State, Bloemfontein, South Africa; 6 Centre of Excellence for Nutrition (CEN), North-West University, Potchefstroom, South Africa; 7 Department of Paediatrics and Child Health, Red Cross War Memorial Children’s Hospital, University of Cape Town, Cape Town, South Africa; 8 MRC Lifecourse Epidemiology Centre and NIHR Southampton Biomedical Research Centre, University of Southampton and University Hospital Southampton NHS Foundation Trust, Southampton, United Kingdom; General Hospital Korgialenio Benakio, GREECE

## Abstract

Individuals’ lifestyle behaviours determine health. Improving Early Nutrition and Health in South Africa (“ImpENSA”), an EU Erasmus+ co-funded project, aims to tackle the triple burden of malnutrition in South Africa through equipping healthcare professionals (HCPs) with knowledge and skills to effectively support healthy nutritional choices among pregnant women and mothers/infant caregivers. Healthy Conversation Skills (HCS) is a behaviour change intervention utilising open discovery questions, active listening, reflection on practice and goal-setting support through SMARTER (Specific, Measured, Action-oriented, Realistic, Timed, Evaluated and Reviewed) planning as core competences. We integrated HCS training delivered online (eHCS training) as practical skills training for HCPs following two eLearning modules on micronutrient nutrition and person-centred behaviour change support. In a programme pilot (2021–22) we investigated participant training experiences, application of HCS and changes in clinical practice using assessments, questionnaires, and interviews at baseline, pre- and post-training and at 3-month follow-up. Of 36 participants (mainly dietitians) initially enrolled, 27 completed eHCS training and 24 participated in the follow-up evaluation. All applied open discovery questions and listened to their patients more actively (spending less time giving information) during consultations. Many participants (63%) reported that they frequently supported their patients using SMARTER planning for a behaviour change. All reflected on their practice and how they consulted their patients. Participants gave very positive feedback on eHCS training, finding it relevant and enjoyable. Contextual (micronutrient nutrition) and theoretical (person-centred behaviour change and HCS) knowledge established prior to eHCS training enabled participants to focus during the training on practising and mastering HCS and observing others. This facilitated reflection, deepened understanding of patient-centred care and accelerated the application of HCS to implement person-centred care in their practice. We conclude that eHCS training delivered online, integrated with knowledge-based modules, can effectively enhance the way HCPs support their patients to ultimately optimise early life nutrition.

## Introduction

The World Health Organization (WHO) states that “*Individual behaviour and social circumstances together account for 60% of factors determining people’s health*” [1]. This highlights the importance of addressing human behaviour to improve outcomes related to longstanding public health problems, such as the increasing prevalence of overweight, obesity and non-communicable diseases [2]. In particular, lifestyle behaviours, such as unhealthy dietary choices or low levels of physical activity, have well documented adverse effects on health [3–5]. While diet is one of the leading determinants of health across the life course, it is particularly important in the period from preconception to infancy and early childhood (the first 1000 days), a critical time of vulnerability to adverse environmental exposures [6]. An excessive or insufficient nutrient supply due to poor quality of maternal diet during pregnancy, suboptimal breastfeeding or complementary feeding practices in infancy, which are common in low- and middle-income countries such as South Africa, can negatively affect child growth and development and have lasting consequences on health. High-quality health services that draw on behavioural and cultural insights can effectively improve individual’s actions or/and habits affecting their own health and that of the public [7]. Healthcare professionals (HCPs) play an important role in supporting their patients and clients to improve their health behaviours. However, healthcare practice in South Africa, including nutrition counselling, often follows a traditional instructive approach, which is ineffective in facilitating sustained behaviour change [8]. The World Health Organization recognises person-centred care as a key component of developing high quality healthcare [9]. It has been proven to be effective, especially for conditions that require lifestyle and behaviour changes [10]. HCPs take into account clients/patients’ values, preferences and needs, actively involve them in health-related decision processes and support them to achieve a desired behavioural goal [11]. Person-centred care has proven to be beneficial in relation to health outcomes, and patients appreciate individualised care and information [12]. However, barriers make it difficult for HCPs to shift from an expert-led, instructive approach towards person-centred care. Lack of adequate behaviour change training, which facilitates the acquisition of skills that can be applied with confidence in practice, is one of the frequently reported barriers [8].

Behaviour change interventions that enhance self-efficacy, such as Healthy Conversation Skills (HCS), have been shown to positively affect health behaviours and improve health outcomes in various clinical settings and contexts across the world [13–15].

### Healthy Conversation Skills (HCS)

HCS was developed by a multi-disciplinary research team at the University of Southampton, with expertise in psychology, nutrition and public health, supported by staff from Southampton City Council and National Health Service, United Kingdom. Briefly, the HCS approach is based on the following philosophies: i) I am not responsible for the choices people make; ii) Being given information alone does not make people change; iii) People come to us with solutions; iv) It is not possible to persuade people to change their habits. The core skills include open discovery questions (ODQs), active listening, reflection on practice, and goal-setting support through SMARTER (Specific, Measured, Action-oriented, Realistic, Timed, Evaluated and Reviewed) planning. The details of HCS, including its theoretical design background, are described elsewhere [16].

### Electronic Healthy Conversation Skills (eHCS) training

HCS training, delivered face-to-face, aims to equip HCPs with skills to effectively support behaviour change of their patients/clients through increasing their self-efficacy–supporting patients/clients to identify solutions by themselves and thus be more likely to achieve intended behavioural goals. Further details of HCS training are described elsewhere [17].

The COVID-19 pandemic limited the delivery of face-to-face training. To overcome this challenge and continue to deliver HCS training globally, the HCS development team redesigned the training activities for online delivery (eHCS training), while keeping the purpose and integrity of each activity as originally intended. eHCS training utilises the Zoom platform and its tools, including the chat function, breakout rooms and annotation tool to facilitate active engagement between trainer(s) and participants, and between participants. Two trainers/coordinators, one acting as a technology host and the other as a facilitator, deliver eHCS training in an interactive and engaging manner, modelling HCS philosophies and skills.

### ImpENSA project and training programme

The Improving Early Nutrition and Health in South Africa through capacity building (ImpENSA) project is an EU Erasmus+ funded multi-country initiative coordinated by LMU Munich. It aims to tackle the triple burden of malnutrition in Southern Africa. The project consortium has 8 European and South African partners (Ludwig-Maximilians-University, Munich, Germany; University of Southampton, United Kingdom; Medical University of Warsaw, Poland; and North-West University; Stellenbosch University; University of Cape Town; Association for Dietetics in South Africa; and Nutrition Society of South Africa). The project’s goal is to substantially enhance the knowledge and behaviour change support skills of HCPs in relation to the nutrition of mothers and their babies. To achieve this, it developed an innovative, evidence-based, scalable training programme, called “ImpENSA Training Programme”, for HCPs, focusing on the importance of micronutrient nutrition during the first 1000 days and evidence-based methods of supporting behaviour change.

The training programme consists of three modules–two self-directed eLearning and one facilitated practical skills modules (the programme and module outlines are shown in S1 Fig). The first eLearning module (Module 1) is about micronutrient nutrition in the first 1000 days, growth, development and nutritional programming; the second eLearning module (Module 2) is about behaviour change and effective communication to facilitate and support behaviour change; and the facilitated practical skills module (Module 3, eHCS training) is about practising and mastering HCS. After completing the ImpENSA Training Programme, we expected that HCPs would become more patient-centred and be able to change the way that they supported patients and that the positive outcomes experienced from making the changes would encourage them to continue making changes.

We piloted the training programme to investigate its effectiveness, and here we report the findings from this pilot study, specifically with respect to eHCS training.

## Materials and methods

### Study design

We conducted a pre-and post-intervention study to investigate the effectiveness of the ImpENSA Training Programme. The study was reviewed and approved by the Human Research Ethics Committee of North-West University, South Africa (NWU-00259-21-A1) and Bioethics Committee of the Medical University of Warsaw, Poland (AKBE/114/2020, KB/52/A2021). Recruitment of study participants took place between 8 October 2021 and 24 January 2022. All study participants provided written informed consent. Due to COVID-19 restrictions, both training and evaluation activities were carried out online.

We applied Kirkpatrick’s model (four evaluation levels: Reaction, Learning, Behaviour, and Results) [18] as a theoretical framework underpinning eHCS training evaluation [16].

### Integration of eHCS training in the ImpENSA Training Programme

**Fig 1:**
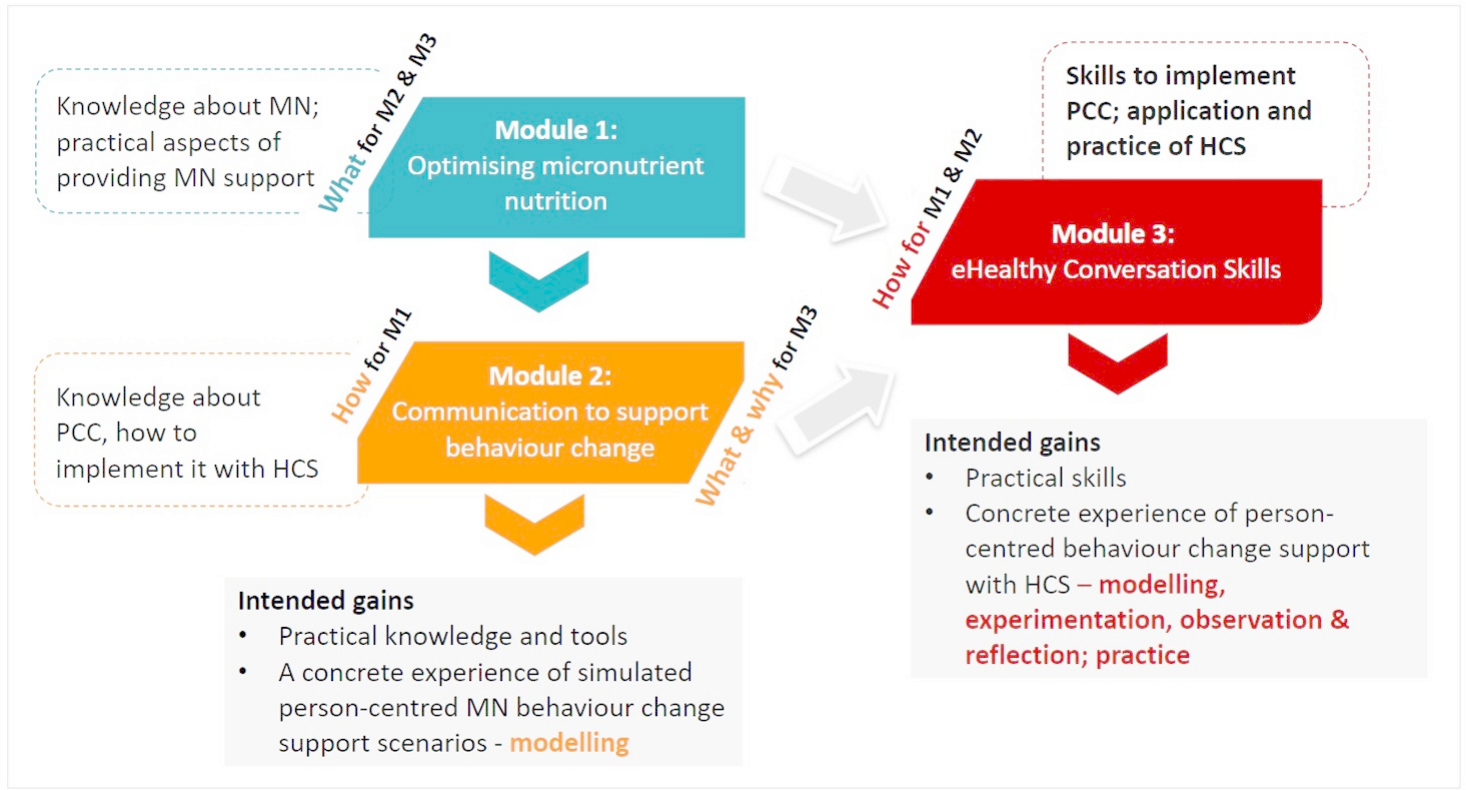
eHCS training integrated into ImpENSA Training Program–schematic presentation (M1: eLearning module on micronutrient nutrition, M2: eLearning module on behaviour change support, M3: eHCS training, MN: micronutrient nutrition, PCC: person-centred care).

Integrated with eLearning modules that provide relevant (clinical/work) contextual and theoretical knowledge bases on micronutrient nutrition and person-centred behaviour change support, eHCS training (Module 3 practical skills training) was intended to enhance HCPs’ communication with, and care provided to pregnant women and mothers/caregivers of infants and young children (Fig 1).

HCS training contextualises activities around participants’ own experiences, recognising the diversity in their backgrounds, but also focuses on common lifestyle behaviour changes such as improving diet and physical activity levels. eHCS training within the ImpENSA Training Programme was tailored to optimise micronutrient nutrition during the first 1000 days. This was reflected across training activities, where real-life nutrition/dietetic consultation scenarios and shared stories related to pregnancy, breastfeeding and complementary feeding practices in South African contexts were incorporated, and these were aligned to the content of the eLearning modules. In addition, new activities were introduced, such as using HCS for effective feedback, practising critical reflection, and making “If-Then” plans, to ensure participants were more likely to implement newly gained skills in practice after the training (S1 Appendix).

### Participant selection and procedures

HCPs from South Africa, namely registered dietitians, nutritionists, nurses and midwives, who provided care to pregnant women or mothers/caregivers of infants and young children with at least 2 years’ work experience and access to the internet, were eligible to participate in the study. Study participants were recruited through newsletters from the Association for Dietetics in South Africa and networks of project consortium partners. LHN and EV (North-West University) communicated with the HCPs who expressed interest in the study, assessed their eligibility for the study participation, and obtained written informed consent from those meeting the eligibility criteria. Of 40 HCPs who expressed interest in the study, 36 met the eligibility criteria and were enrolled in the study.

The ImpENSA Training Programme was delivered to four groups; the number of participants per group ranged from 6 to 11. Upon creating a user account on the training platform, participants were invited to complete baseline evaluation activities scheduled in a 1.5-hour Zoom meeting. Questionnaire and assessment took approx. 30 minutes each. Afterwards, they were given access to the eLearning modules to complete, which was a prerequisite for eHCS training.

eHCS training was delivered by three newly trained South African coordinators (LHN, EV and one non-project partner at Stellenbosch), who had completed an online trainer programme called ‘Train-the-Coordinator Mentoring Programme’ specifically designed to train new ImpENSA eHCS coordinators. One coordinator (LHN) facilitated training for all four groups, in a pair with one of the other two coordinators. For three training groups, eHCS training was delivered within 1–2 weeks after the completion of eLearning modules; for one group, this period was extended to two months to accommodate the seasonal holidays in South Africa. eHCS training, consisting of two 4-hour sessions, delivered a few days apart to each training group provided an opportunity to practise newly gained skills between the sessions.

**Fig 2:**
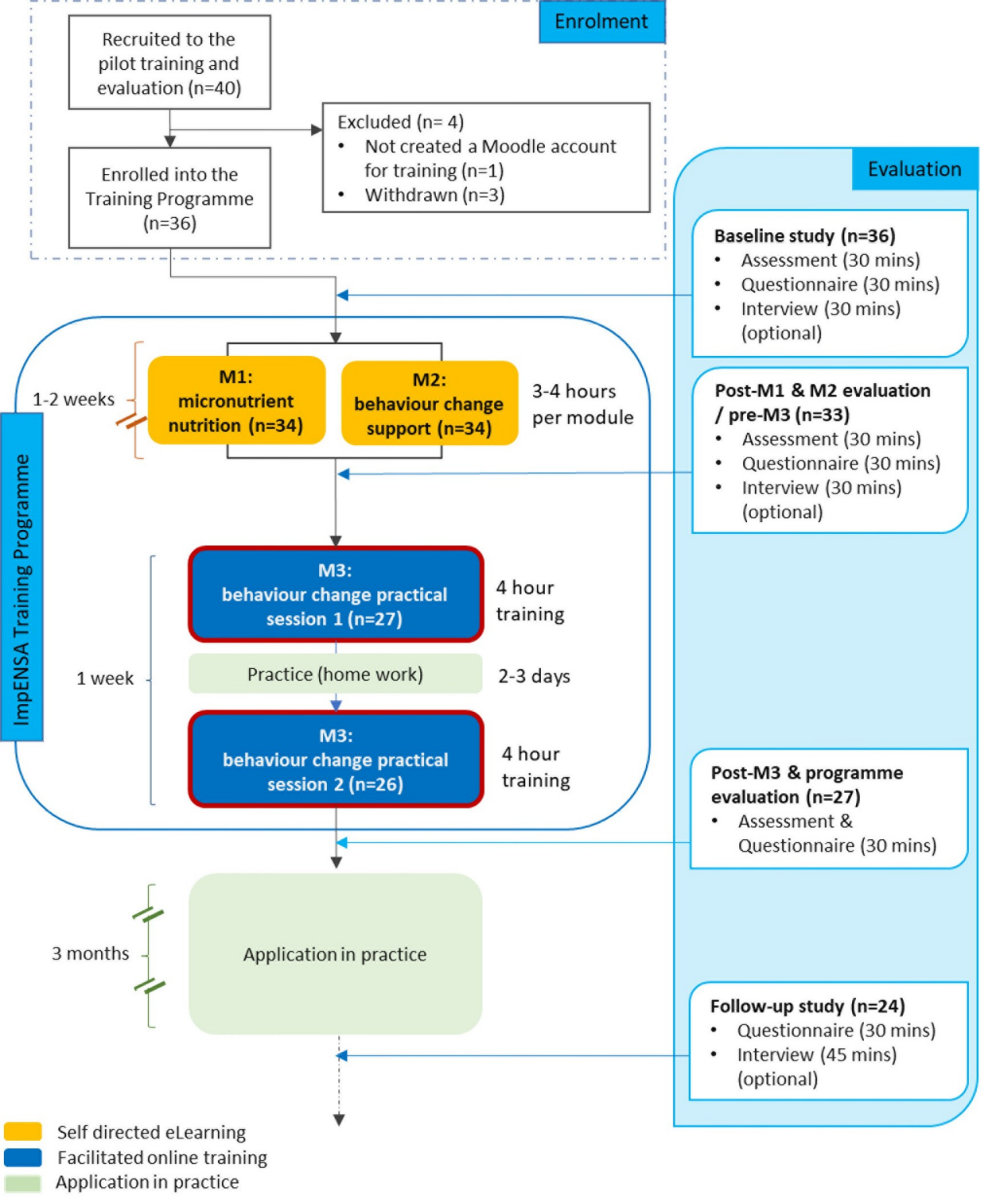
ImpENSA Training Programme pilot study flow chart (M1: eLearning module on micronutrient nutrition, M2: eLearning module on behaviour change support, M3: eHCS training).

Post-eLearning modules/pre-eHCS training an assessment was carried out online. Post-eHCS training participants were also invited to complete a questionnaire and an assessment. Three months after the training, participants who completed the training were invited to participate in the follow-up evaluation, comprising a self-administered online questionnaire and an optional interview. For details on all training and evaluation activities, including evaluation of eLearning modules, see Fig 2.

Follow-up interviews were conducted on the Zoom platform in English by two researchers (BPG, a paediatrician with an interest in early nutrition, conducting an interview and SCh, education researcher and learning designer with an interest in nutrition capacity building, taking field notes and asking additional prompt questions). Both researchers had earlier experience of HCS training themselves and had observed multiple eHCS training sessions. Interviewers obtained participant consent for recording and conducted an interview (of approximately 45-minute duration) that covered the evaluation of eHCS training as well as the overall ImpENSA Training Programme and eLearning modules.

### Data collection and outcomes measures

We evaluated eHCS training using quantitative and qualitative methods to ensure triangulation of the results. For assessments and questionnaires, we adopted the established HCS training evaluation tools [15, 17, 19, 20], adapting the content to be appropriate for the evaluation of the eHCS training integrated in the ImpENSA Training Programme (S2 Appendix). Additional questions for a follow-up questionnaire and an interview guide (S3 Appendix) were developed. The tools afterwards were reviewed initially by the project evaluation team and followed by the project steering committee to ensure the tools were appropriate to assess the effectiveness of the training programme in South Africa. Utilising these methods and tools we investigated: i) skills gain ‐ ability to formulate and ask ODQs, ii) application of HCS in practice at follow-up, iii) confidence gain in supporting patients to make a behaviour change in relation to early nutrition, iv) other outcomes, v) reaction to eHCS training and training features that facilitated skills gain and application in practice.

#### Skills gain ‐ ability to formulate and ask ODQs

An assessment measured participants’ ability to use ODQs at three time points (baseline, pre- and post-eHCS training) and changes over time–the progression of skills development over the course of the ImpENSA Training Programme (eLearning modules and skills training). Participants were asked to write their responses to four quotes (i.e. what they would say to support a pregnant woman or a mother/caregiver of an infant to make a behaviour change in relation to their diet and health or breastfeeding and complementary feeding practice). These responses were later coded into 7 categories.

#### Application of HCS in practice at follow-up

Closed questions (i.e. Likert-scale and Yes/No questions) in the follow-up questionnaire investigated the application of HCS in practice (ODQs, active listening, SMARTER planning, reflective practice) after completing the training, including the frequency/intensity and purposes of using HCS. In the follow-up interviews, we explored how participants utilised HCS in consultations, what the purpose of using HCS was, and how the use of HCS had changed after the training. Participants were prompted to describe how they used each skill with a recent consultation as an example.

#### Confidence gain in supporting patients to make a behaviour change in relation to early nutrition

Ten-point Likert scale questions in the baseline and follow-up questionnaires assessed participant confidence in supporting patients to make a change in relation to micronutrient nutrition using HCS at each time point and changes over time.

#### Other outcomes

In a post-training questionnaire and during follow-up interviews we explored other outcomes that emerged from the ImpENSA Training Programme (eHCS training contributed to) and associated evaluation activities.

#### Reaction to eHCS training and training features that facilitated skills gain and application in practice

A post-eHCS training questionnaire explored participants’ experience with the training: a 5-point Likert scale question on their overall eHCS training experience; and open questions for training features and aspects participants found particularly helpful and enjoyable, and suggested improvements. During follow-up interviews, we further explored features of eHCS training that had helped participants to apply HCS and make changes towards person-centred care in their practice.

### Data analysis

Descriptive statistics were used to present the demographic characteristics of participants. Ordinal data in the paired samples (i. e. Likert scale questions repeated at different time points), with no clear normal distribution were analysed with Wilcoxon signed rank tests. Responses to the quotes in the assessment were coded using a previously developed coding rubric, with scores ranging from 0 to 7 [15, 17, 19]. All responses were double-coded by two researchers (BPG, SCh) to ensure coding consistency. Any discrepancies were resolved by consulting WTL (Health Psychologist, member of the HCS development team and HCS Super Trainer). The categories assigned to quote responses were compared between 3 time points (at baseline, pre- and post-eHCS training) with McNemar’s tests of categorised responses dichotomised as follows: ODQ response (category 6–7) and other responses (category 0–5).

All statistical tests were two-sided, with 5% threshold of significance. For the questions asked at only one time point during the evaluation, the results were presented descriptively. The data were analysed with StatsDirect software (v. 3.3.5). Interviews were audio-recorded and transcribed verbatim. Thematic analysis of follow-up interview transcripts and open questionnaire comments was performed independently by two researchers (BPG, SCh) to explore outcomes resulting from eHCS training and participants’ experience with the training. Either ATLAS.ti Software (Version: 22.2.0.225 (23.11.2022 12:56:17)) or Nvivo software (QSR International, Version 12) was used for this analysis.

### Inclusivity in global research

Additional information regarding the ethical, cultural, and scientific considerations specific to inclusivity in global research is included in the Supporting Information (S1 Checklist).

## Results

Twenty-six participants completed and one partially completed eHCS training and took part in the eHCS training evaluation activities at baseline, and pre- and post-eHCS training (S1 Table). Three months after the training, 24 of these participants (92%) took part in the follow-up evaluation study.

### Characteristics of participants

All 27 participants were female, mostly dietitians (96%) and working in an urban setting (85%). The proportions who worked in the public and private sectors were similar (44% and 56%, respectively). Participants mainly provided their services in community health centres (33%) and hospitals (33%), while some worked in private practice (15%) or in multiple organisations (11%).

### HCS skills gain–ability to formulate and ask ODQs

**Fig 3:**
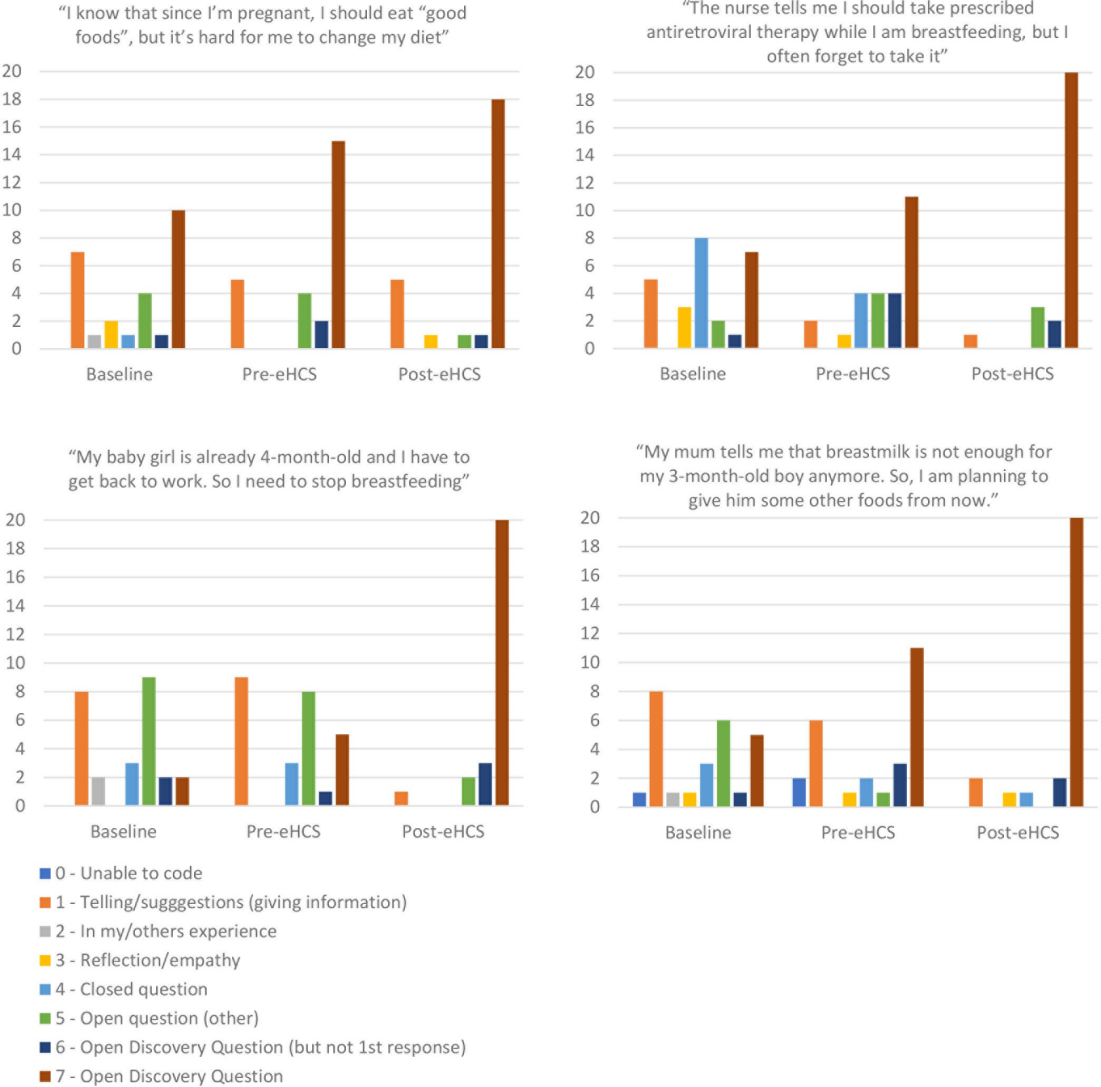
Response categories provided by participants to four written quotes about early life health behaviours at baseline, pre- and post-eHCS training.

Post-eHCS training, 83% of all responses provided by participants (n = 26) to four quotes about early life health behaviours were formulated as ODQs (response categories 6 or 7) compared to 28% at baseline (p<0.001). Categories of participants’ responses to individual quotes are presented in Fig 3, which shows the same pattern of changes in responses for all individual quotes. Participants’ ability to formulate and ask ODQs gradually improved during the ImpENSA Training Programme, with significant differences (p<0.001) observed between baseline and pre-eHCS training and between pre-and post-eHCS training, indicating the role of both eLearning modules and eHCS training in skills acquisition.

### Application of HCS in practice at follow-up

#### ODQs

At follow-up, 23 participants (96%) agreed (or strongly agreed) with the statement ’*I have used Open Discovery Questions (‘what’ and ‘how’ questions) more often when supporting my patients to make a behaviour change*’. Participants primarily used ODQs to explore patients’ environments to identify barriers and facilitators for a behaviour change and help patients identify solutions that were appropriate for their circumstances (S2 Table). ODQs were also applied to SMARTER planning, with 18 participants (76%) reporting using ODQs often or always when supporting their patients in making, evaluating and reviewing a SMARTER plan.

*…my consultations was, before ImpENSA is that I would gather all the information in the first half of the session and in the second half of the session I would give them feedback or I would tell them what to do in the second half of the session. And now my sessions have changed from that. When they start entering my office, I ask them open-ended questions and we discuss what their concerns are just there and then and then I go onto the next concerns, the next concerns and it feels to me, like, when they leave here, I really addressed their concerns much better than when I leave it for the end and just give them the feedback. Yeah.* [P43_Dietitian_Private_Int]

… *in the beginning [before the training] I would say*, *“I would like you to do this and this and this*.*” And now*, *I’m asking*, *“How do you think you want to achieve this goal*? *Or what is it that you want to achieve*? *Or by when would you like to achieve this*?*” So*, *instead of me saying*, *“This is what I want you to do*, *and then by four weeks*, *I’m expecting these results*.*” So*, *definitely open-ended questions*. [P47_Dietitian_Private_Int]

#### Active listening

At follow-up, 23 participants (96%) also agreed (or strongly agreed) with the statement, ’*since completing the ImpENSA training*, *I listen to my patients more and spend less time than previously on giving information or making suggestions to my patient*s’. While the proportions of consultation time spent on listening differed between participants and between initial and follow-up consultations, for many, listening accounted for more than 50% (up to 90%) of a whole consultation.

*“…before, I would spend a short time asking questions to get background information and a lot of time doing the counselling and getting the information out there whereas now, the feedback is more specific because it’s based on what the patient has said. In order to get that, you need to listen, you need to actively listen and pay attention to the patient otherwise, you’re going to end up giving generic information anyway. I think I have shifted a lot more to patient specific counselling, so that means that I would have to listen to the patient more. But also, with asking all these open discovery questions, it’s kind of pointless if you don’t listen or actively listen. So, it kind of goes together but I think I do it more.* [P05_Dietitian_Private_Int]

#### SMARTER planning

The frequency of using SMARTER planning in consultations and how participants (n = 24) used it varied. While a proportion of participants (17%) indicated supporting their patients in making a SMARTER plan for a behaviour change always, the majority (75%) used SMARTER planning often or sometimes (46% often, 29% sometimes), with a small proportion of participants (8%) using it rarely.

*“So that it’s more of a patient-driven approach, instead of me just talking, me listening a little bit more and to find out as much as possible from the patient before kind of interfering and me leading into the goals. rather let the patient lead the goals or set them. Yeah, I’m using the SMARTER objectives definitely to make sure that it’s their goals that are achievable and not just out there. Okay, you want to lose 20 kilograms after your pregnancy, that’s all good and well. But how do we get there? And let the patient drive that instead of me driving that, yeah."* [P75_Dietitian_Private_Int]

Some used the SMARTER planning tool step-by-step but the majority applied SMARTER principles, using the tool as a guide. Types of care participants provided to patients and patients’ needs appeared to have influenced these differing frequencies and use of the SMARTER planning tool. SMARTER planning was used frequently in consultations with patients who required dietetic treatments, for example as part of the treatment of infertility and chronic conditions, and in follow-up consultation. It was used less frequently for patients/clients who did not require dietetic treatments, for example healthy breastfeeding mothers, or patients who sought expert advice, and in initial consultations.

*“So, while the SMARTER acronym is valuable in its core, I don’t use it as the acronym itself*. *I would use the different … Because some situations, clients jump over some of them or already incorporate some of the other aspects into their goal setting. So, for the goal setting, specifically, I use SMARTER principles to make sure we’ve got everything in there. But then also for my own practice, I try to reflect back on the SMARTER techniques and try to improve my own practice looking at those.” P74*

*“So*, *that obviously is with your follow-ups*. *So*, *I would ask*, *“How did you manage with your goals*? *What do you think were challenges in achieving your goals*?*” And then we would*, *obviously*, *from that reflect on what worked and what didn’t work*. *And then*, *depending on what the issue were*, *we would set up another SMARTER goal*.*”* [P47_Dietitian_Private_Int]

In addition, participants’ familiarity/mastery of SMARTER planning influenced their application of it in consultations. Several participants reported having had a conceptual understanding of SMART planning/goal setting before the pilot study but had not applied it in practice.

*“Before the training, I didn’t use the SMARTER goals at all. It wasn’t new to me. I did know about it, but I never saw the practical application in my situation. That was a big strategy that I implemented to use with the patients because they had set out very clearly the steps throughout reaching the goals, and that is, it’s helped me a lot. It helped set structure automatically without me even having to set out that structure. That helped me a lot.”* [P11_Dietitian_Public_Int]

For participants who were new to the concept, implementing SMARTER planning in practice required more effort, time and practice with some seeing this as a work in progress. A few participants mentioned that an additional training session to practise it would help familiarise them with using SMARTER planning.

*“And then definitely the SMARTER goals setting was very, very helpful. I basically just printed out the attachment [SMARTER plan tool] that you guys provided for us and folded in. I must say that it does take some time. It’s quite a hard thing to actually fall in. And it’s very hard for the client to come up with things, as well, specifically with that. But I think it’s because sometimes it feels like we are repeating ourselves with the SMARTER goal. So, I’m still trying to figure that out. But I do find it very, very helpful in helping the client realize what they actually want to do and how motivated they are to actually do it, yeah.”* [P24_Dietitian_Private_Int]

#### Reflective practice

All study participants (n = 24) agreed/strongly agreed that since completing the ImpENSA Training Programme, they reflected on their practice and the way they consulted their patients more often. Several applied SMARTER principles to review and reflect on their practice.

*I probably reflected on how I originally conducted my consultations versus now. I think it has forced me to kind of re-evaluate after every patient just to see that did that work, what can I do better, was it successful? Every patient is different, so you have to kind of reflect after every consultation because not everyone would require the same management but I mean, the background [Inaudible 00:12:12] is obviously the same but it’s always good to remind yourself of what you can do better or how you could change something. I think especially when changing from your old style of consulting to your new style because you need to remind yourself of the things that you used to do and what you should do better.* [P05_Dietitian_Private_Int]

*But then also for my own practice*, *I try to reflect back on the SMARTER techniques and try to improve my own practice looking at those*. *So*, *while I’ve got the acronym on the wall*, *important to be reminded of them because if it’s not* … *So*, *it’s now in my consultation*, *it’s in my private wall at home*, *so that I can be reminded of this*. *So*, *the more I practice it on looking into my own behaviour*, *the easier it becomes to ask these questions and prompt them based on the principles of the SMARTER acronym*. [P74_Dietitian_Private_Int]

### Confidence gain in supporting patients to make a behaviour change in relation to early nutrition

**Fig 4:**
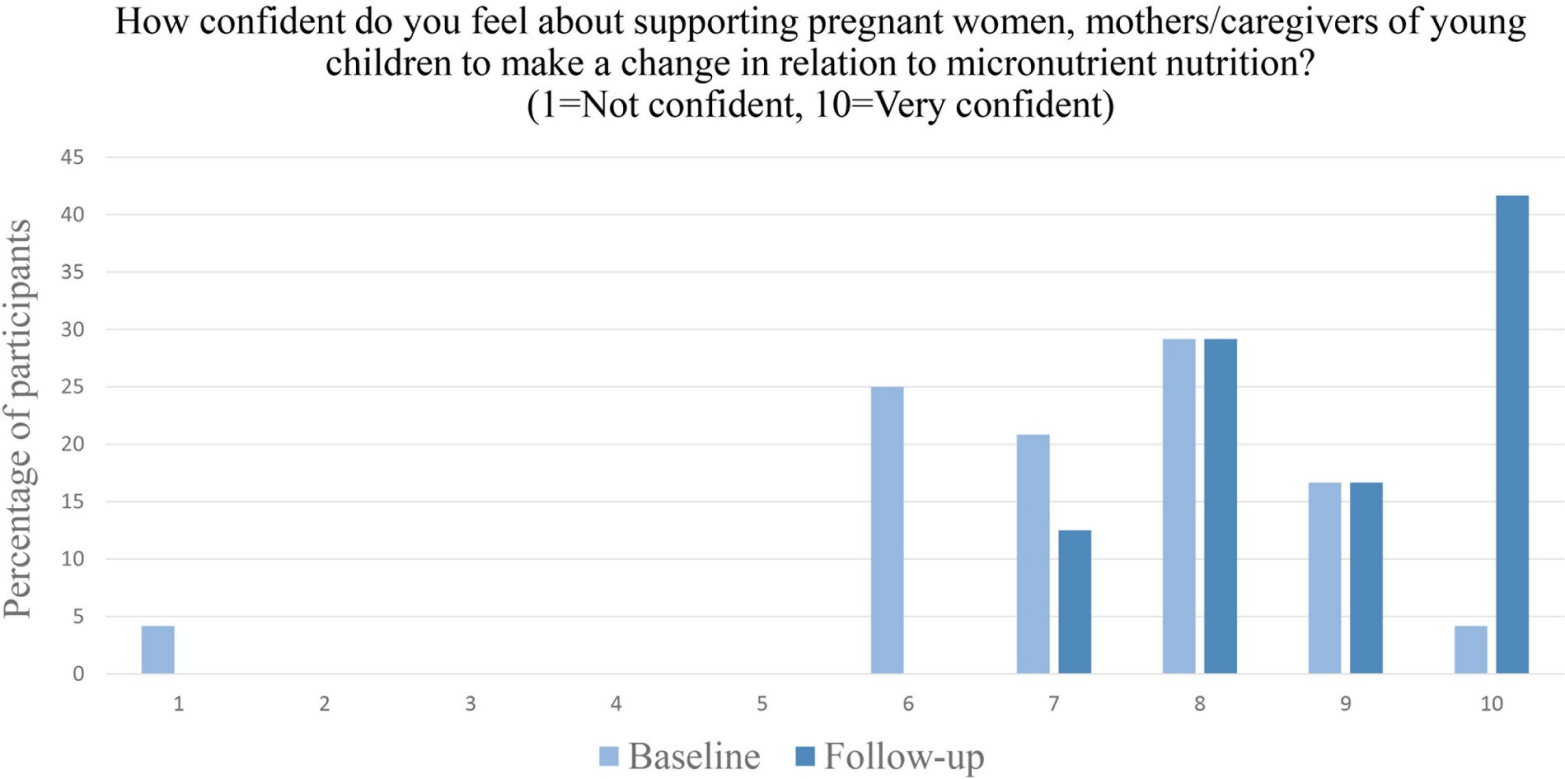
Participants’ confidence in supporting patients to change a behaviour (in relation to micronutrient nutrition), n = 24.

From baseline to follow-up there was a significant shift from participants feeling moderately confident in supporting their patients to make a behaviour change (in relation to micronutrient nutrition) to feeling very highly confident (p <0.001) (Fig 4).

Participants’ improved confidence in supporting their clients in making a behaviour change was attributed to increased knowledge and skills to provide patient-centred nutrition care about micronutrient nutrition and person-centred care, along with the practical skills to deliver/implement person-centred care. Participants, who had an understanding of person-centred care from previous training but were not able to fully implement it due to a lack of skills/techniques to do so particularly appreciated the role-modelling and practical nature of the eHCS training.

*“Like I mentioned, module one improved my confidence with the information that you need to give the mom, but especially module three was just the way how I should be working with the mom, so that improved my confidence with the skills part.”* (P48_Dietitian_Public_Int)

### Other outcomes

Other outcomes participants reported in the post-questionnaire and during the follow-up interviews were as follows.

#### Attitude/ mindset change

Participants reported gaining a better understanding of person-centred care and why it is important, which subsequently changed their views on patient care and their role in supporting patient behaviour change. Participants perceived the approach to behaviour change support to be teamwork between HCPs and patients with patients taking the responsibility for making changes [21]. Attitudes and mindset changes towards person-centred care and their roles were gradual, initiated from learning about and experiencing person-centred care in eLearning modules and deepened through practising it in eHCS training followed by its implementation in their own practice.

*“I think the biggest take back from the training for me is having the ability to place yourself in your patients’ shoes, and acknowledging how difficult behaviour change actually is. Also that resistance to change is influenced by so many things, that if you took the time to actually properly investigate by asking the right question (and also in the right way, like open discovery question) you will have a better understanding of your patients, their individual struggles, and their self-identified strategies to overcome them.”* [P26_Dietitian_Public_Quest]

*“I must say prior to the session*, *you would feel that it’s your responsibility*, *and the client’s behaviour change is a direct reflection of your efforts*, *but I really now feel that you need to provide as much support and education as needed*. *But overall*, *it is the client’s behaviour that needs to change*, *and they are responsible for that*. *So that’s a big thing*.*”* [P48_Dietitian_Public_Int]

#### Altered consultation style with a shift towards person-centred care

Participants reportedly changed their approach to the care they provided to patients/clients from prescriptive (telling/giving information) to more person-centred. They moved away from providing generic advice to more personalised, holistic support, tailored to meet individual needs [21].

*“I think previously, it was a generic thing. It was more about ticking the boxes, telling the patients about it because every patient needs to know about it whereas now, it’s more patient specific. If this patient only wants to know or if I gather from the question that they’ve kind of got the micronutrients done but they’re struggling with the side effects of iron for example, then we would speak about that. I think it’s more specific now rather than just spitting out general information from the [South African] booklet or whatever the case may be because the patient needs to know it.”* [P05_Dietitian_Private_Int]

Participants experienced positive results from shifting their consultation style towards person-centred care; reported benefits included open relationship and better engagement with patients, increased patient motivation to change, improved patient outcomes, improved job satisfaction and confidence, and personal and professional development [21].

### Reaction to eHCS training and training features that facilitated skills gain and application in practice

Overall participant experiences with eHCS training, assessed directly after the training, were very positive (n = 26, mean score 4.46, SD 0.57 on the 1–5 scale with 1 being the lowest and 5 the highest level). This was echoed in participants’ open comments in the post-eHCS training questionnaire and 3-month follow-up interviews, most saying the training was “*very informative"*, *" really enjoyed" and "useful"*.

We analysed post-eHCS questionnaire open comments and follow-up interview transcripts to identify the features/aspects of the training that participants found most useful and enjoyable and that participants considered would enhance their implementation of person-centred care using gained HCS. We developed 8 themes: 1) relevant and skills-focused training with tools to use, 2) active participant engagement, 3) hands-on training, 4) establishing contextual [micronutrients nutrition] knowledge base before practical training, 5) establishing theoretical [person-centred behaviour change support] knowledge base before practical training, 6) technology enabled/enhanced delivery, 7) follow-up support, and 8) practising HCS use in a whole consultation and more opportunities to practise.

#### Theme 1: Relevant and skills-focused training with tools to use

One major motivation for participants taking part in the ImpENSA Training Programme pilot study was to improve the behaviour change support they provided to patients in order to improve patient outcomes and follow-up [21]. Participants appreciated the skills-based nature of eHCS training (opportunity for skills based-training in person centred care is rare compared to knowledge-based training) as this helped them to implement the skills in their clinical practice. Participants had become familiar with the concept of person-centred behaviour change support and were keen to change their practice to this approach after the eLearning modules. eHCS training enhanced their understanding of the approach, helped them practise and master HCS, and prepared them to implement the skills in practice.

*“So, I felt like, after we’ve done the training and we’ve practised on each other, it was so practical, I could immediately start using it in practice where I would previously have to go and summarize it and kind of find a way to start using this in the practice, where now, I remembered it and it was something I could just apply.”*[P22_Dietitian_Private_Int]

*“And then when I did the course*, *when I got the little template of the SMARTER goals*, *I was like*, *“Oh*, *this is so cute because there’s questions but I can actually ask the person*.*” And I can actually pick and choose which questions I can use based on the person sitting in front of me and what they need at this moment*. *So*, *that really helped*, *yeah*.*”* [P24_Dietitian_Private_Int]

#### Theme 2: Active participant engagement

One of the training features appreciated by most participants was its highly interactive nature, promoting active engagement between the facilitator and participants and between participants. Activities, such as facilitators interacting with participants using ODQs (modelling), paired activities or role playing (simulated experience) actively engaged participants in the training. Participants valued the training providing a platform for discussion between a facilitator and participants and an exchange of experiences and ideas between participants themselves, which facilitated reflective and critical thinking. By supporting the interaction between participants who were in the same nutrition profession, the training additionally strengthened their sense of belonging to a professional group who faced similar challenges and raised an awareness of different consultation styles, prompting reflection.

*“Facilitators were very knowledgeable, they created an environment where we could feel free to ask questions and I personally enjoyed the discussion aspect of the training. They used clever techniques and exercises to keep us engaged and our minds constantly thinking.”* [P04_Dietitian_Public_Quest]

*“It was very engaging*, *and the examples and activities given made it easy to see the practical application*. *Very nice to have different partners*, *to speak to a lot of people*. *We could also see then how others would ask a similar question in a better way*.*”* [P11_Dietitian_Public_Quest]

#### Theme 3: Hands-on training

Participants liked “hands-on” activities in the training. They experienced the theory (person-centred behaviour change support) and how HCS could be used in practice. They were able to practise the skills in the training during which they also had an opportunity to observe, reflect, actively experiment and (re)conceptualise along the process of mastering the skills. This supported and accelerated their implementation of HCS in their clinical practice during and after the training.

*“So, I definitely would say the practical side of things, all the practical activities we were doing because I felt like, when we just did the training with module two, I wasn’t that comfortable with the open-ended questions and the listening until we started to do the activities and we kind of practiced on each other. So, then I got more comfortable using it in the practice, to be able to now continue to use it after.”* [P22_Dietitian_Private_Int]

*“The activities definitely helped quite a lot because it’s easy just to read something but to actually then practice it is a different story*. *To be able to practice it with other people that are also busy with the pilot study*, *that really helped a lot because that builds your confidence to actually be able to do it with a patient*. *Yes*, *I definitely found that very useful*.*”* [P51_Dietitian_Private_Int]

#### Theme 4: Establishing contextual [micronutrients nutrition] knowledge before practical training

Practising behaviour change support in the same clinical context (micronutrient nutrition) with authentic relevant tasks helped participants experience how to use HCS in practice. Participants were able to observe how others interacted with their patients/clients (in the training and in their practice) and learnt from each other. Most participants in the present study were dietitians and they already had some knowledge about micronutrient nutrition at baseline. Module 1 helped participants to refresh, update and restructure their theoretical knowledge to be usable in practice and provided a common focus, as well as a contextual topic area in which the skills (HCS) were practised.

*“And I really enjoyed that because you learn from other people and you see that you’re not alone, or you get ideas from other people based on what you’ve learned in the course* [P03_Dietitian_Public_Int]

*“Definitely the knowledge recap and there was definitely additional knowledge that I gained in module one and two*, *that also just gave me additional confidence to really treat my patients*.*”* [P48_Dietitian_Public_Int]

#### Theme 5: Establishing theoretical [behaviour change] knowledge base before practical training

Participants reported that the theoretical aspects of person-centred behaviour change support and HCS introduced in Module 2 helped them form a knowledge base and enabled them to focus on practising and mastering the skills in and around the eHCS training sessions. It also prompted them to review and reflect on their practice and plan changes. These attributes consequently accelerated the implementation of the person-centred approach using HCS in their clinical practice.

*“I think it [Module 2 and eHCS] works well together … because of module two, you focus more on the barriers [in the eHCS training], and I think that really provided us with the adequate information to be able to do module three.”* [P48_Dietitian_Public_Int]

#### Theme 6: Technology enabled/enhanced delivery

Technology-enabled learning can widen access to training to those who would not be able to attend it in person. Many appreciated HCS training being made available online (eHCS training). In addition to making it possible for participants to access HCS training during the COVID-19 pandemic, other reasons participants mentioned included: the training connecting many from different geographic locations, reducing the associated cost of travel and time off work, paired and small group activities being facilitated separately with support from Zoom breakout room functions, and simultaneous participation with the “annotation” tool.

*“I think that the online part works really well because it can connect a lot more people.”* [P75_Dietitian_Private_Int]

While the online delivery mode was liked by many participants, some, primarily those working in the public sector, suggested an additional face-to-face option for those lacking internet access and/or preferring in-person interaction.

*“Maybe my only suggestion is maybe possibly making some of it available offline for people in countries that don’t always have access to the internet but that’s not an issue for me.”* [P04_Dietitian_Public_Int]

*“Like if it was in a classroom setting*, *I think would be a lot a lot nicer if there’s like human*, *like extra human interaction unlike doing it online where you break up to the rooms and all that”* [P12_Dietitian _Public_Int.]

#### Theme 7: Follow-up support

Some participants wished to have follow-up activities/meetings to facilitate sharing of further experiences between training participants and consequently help them better implement intended changes in practice.

*“I think it would be it would be nice to have a follow-up session with all the people that we were initially in training with to discuss and see how everybody else is finding it and just discuss. Because without the training, I think everybody did things differently. It would be interesting to see how everybody else also implemented the changes and maybe even pick up more tips on how to improve.”* [P05_Dietitian_Private_Int]

Many considered participation in post-training and follow-up evaluation activities, especially in the interviews, helpful for them to further review and reflect on their practice and the changes they had implemented.

*“What I actually find very useful also, is the conversation afterwards. Like, what are you doing now. Because you don’t always know what you learned and what you don’t learn, you know, what you knew before. And so, a person can easily be arrogant and think you know this because it is new. But it’s actually not the information but it’s the way of changing my way of thinking. With our previous follow ups also, when the questions you asking make me think about it. It feels as if your questions are the same but it forces me to think of my changes that I’m making.”* [P08_Dietitian_Private_Int]

*“And then practicing what we’ve learned the whole time* ***and reflecting back with the interview***. *I think the whole program is really well laid out to be able for us to make the changes and to feel comfortable to make the changes*.*” [*P51_Dietitian_Private_Int]

Some mentioned the provision of a training manual/toolkit that can be accessed offline and referred back to after the training, provided as downloadable materials as a resource to help to consolidate newly gained knowledge and skills.

#### Theme 8: Practising HCS use in a whole consultation and more opportunities to practise

Participants, who were new to the concept of person-centred care and/or were newly qualified dietitians, expressed the need for more opportunities to practise HCS in a facilitated setting. They also found it difficult to apply HCS across a dietary consultation that often requires an educational component, and therefore expressed the need for using scenarios reflecting the entire consultation process in the training.

“*I would rather add more practical case study of one fuller assessment instead of analysing one question and answer. For example how you address the whole consultation. As there are a place for close ended questions.”* [P08_Dietitian_Private_Quest]

*“I think I would have liked a group with the facilitator again to recap on some of the information given*. *Case studies*. *The case studies or even just the examples you gave us if I remember correctly where we had to talk*. *We had breakout sessions and we had to talk*, *and then come back*, *and reflect on how it went or how it was*. *I would like just some more of that*.*”* [P29_Dietitian_Private_Int]

These participants mentioned longer training, a longer time gap between eHCS session 1 and 2, and/or the delivery of the eHCS training in multiple, shorter sessions to allow more opportunities for practising newly gained skills in a real clinical setting.

## Discussion

In this evaluation study we investigated the effectiveness of eHCS training, which was integrated into the ImpENSA Training Programme developed for HCPs, in relation to skills gain, application in practice, and changes made and experienced at their workplace. After the training, participants demonstrated an improved ability to formulate and ask ODQs in response to common/typical patients’ statements referring to early life health behaviours. At follow-up, they reported on changes applied in their clinical practice in relation to key HCS competences, indicating using ODQs more often, spending more time listening to patients rather than giving information, with many utilising SMARTER planning for behaviour change support they provided to patients. Study participants became reflective practitioners, highly confident in supporting patients to change behaviour in relation to early nutrition.

Previous studies on HCS evaluated HCS training as a stand-alone intervention, delivered face-to face, to different groups of HCPs [14, 15, 22]. HCS training proved to be effective when introduced to physiotherapists supporting patients with musculoskeletal conditions [23], primary care practitioners in routine consultations with patients [24], or dietitians supporting lifestyle changes in pregnancy [22]. These previous studies demonstrated that HCS training applied in various clinical contexts and socio-economic settings (high-income or low- and middle-income countries) overall resulted in competence gain and application of HCS in practice among those who completed the training. Our study further supports these findings. However, unlike HCS training in previous studies, in the present study eHCS training was integrated into a training programme additionally consisting of knowledge-based eLearning modules, with the overall aim to enhance behaviour change support provided by HCPs to improve nutrition in the first 1000 days of life. Our findings indicate that after eHCS training, previously equipped with behaviour change theory (Module 2), participants not only acquired and applied HCS in practice, but also developed a deeper understanding of person-centred care. Participants changed their views on patient care and their approach to the patients/clients from prescriptive to more person-centred, outcomes not evaluated and reported before. We know that, implementing person-centred care using HCS reportedly results in open relationships with patients, increased patient motivation to change and improved patient outcomes [25].

The present study supports previous studies where a high level of HCS competency was observed for asking ODQs, active listening and reflecting on practice. However, participants of the previous studies often experienced difficulties in implementing SMARTER planning. This was particularly apparent in a recent study, which assessed the utilisation of HCS after training in South Africa (Soweto) [14]. The authors developed several recommendations for the adaptation of HCS to increase their implementation in South Africa, including simplifying the use of the SMARTER planning tool. They also concluded that the HCS approach may require basic communications skills, which may have been lacking among some community health workers participating in that study. Although the participants in our study were dietitians with a higher education qualification, we believe that differences in the training design, especially eLearning modules providing knowledge bases for micronutrient nutrition (Module 1) and communication to support behaviour change (Module 2) prior to eHCS training, contributed to the greater implementation of SMARTER planning that we observed. Study participants were equipped with knowledge and understanding of person-centred care, which prompted and facilitated review of and reflection on their practice. They intended to implement person-centred care and planned changes even before starting the eHCS training and subsequently applied HCS to do so. Gaining a deeper understanding of person-centred care in eHCS training, supported by direct modelling, experimentation, observation and reflection, likely helped them to use the SMARTER planning tool in a flexible way, responding to their patients/clients’ needs. eLearning modules modelled desired behaviours (person-centred care using HCS) in a real-life scenario resembling their work environment. The process of HCS acquisition in our study started prior to eHCS training, and this resulted in an accelerated overall application of HCS and specifically SMARTER planning, which in itself requires mastery of ODQs and active listening. We believe that this is also highly relevant to reflective practice, already initiated during the two eLearning modules. Experiencing a patient-centred counselling style prompted review and reflection on participants’ own practice, which further facilitated the application of HCS in practice [13]. Finally, we are of the opinion that the significant gain in confidence in supporting patients to make a change in relation to micronutrient nutrition using HCS, reported by our study participants, likely resulted not only from improved behaviour change support skills but also from learning acquired from the micronutrient nutrition eLearning module (Module 1). The module provided up-to-date, evidence-based content on micronutrient nutrition, building confidence in ’what’ participants supported.

As with the original stand-alone HCS training, eHCS training in the ImpENSA Training Programme has several features that are proven to improve nutrition care competence and delivery [26]. These include being relevant for the learner (by meeting their training needs and using real-world examples and scenarios), behavioural modelling through defining and demonstrating desired behaviours by the facilitators, providing participants with opportunities to practise these behaviours, running interactive and engaging activities encouraging experimentation, observation and reflection, and practical techniques and tools. eHCS training in our study additionally offers increased accessibility and enhanced learning through contextualisation in the area of micronutrient nutrition and integration of this topic with theory of person-centred behaviour change, and further integration of self-directed learning (eLearning modules) with facilitated practical skills training (eHCS training). Learning is an active process of constructing knowledge and skills, and for training to be effective, it should be a process of supporting that construction [25]. The feedback we received from study participants suggests that the ImpENSA Training Programme supports this active learning process. In an iterative process it facilitated relevant knowledge and skills (micronutrient nutrition, person-centred behaviour change support and HCS) acquisition and integration, application of gained knowledge and skills in practice, which subsequently accelerated the implementation of person-centred care using HCS. Capitalising on our findings, future training that target capacity building among HCPs to improve care practice could follow the design approach applied in the ImpENSA Training Programme.

### Strengths and limitations

Innovative design and evaluation of the ImpENSA Training Programme, including the effective integration of eHCS training, is an important strength of our study. While quality behavioural counselling interventions have been shown to be effective in supporting healthy behaviours and their importance is widely acknowledged, these interventions are under-utilised in health care [27]. Lack of adequate training for HCPs is an important barrier to filling this gap in preventive health service. In this context, our eHCS training integrated in the ImpENSA Training Programme expands often limited training opportunities in a field of behaviour change support, especially with regard to practical\skills-based training in combination with knowledge-based training. The findings from this evaluation study further confirm that eHCS training was viewed as highly relevant for clinical practice by the study participants. Whilst the training was focused specifically on the context of early life nutrition, the effectiveness of eHCS training in the ImpENSA Training Programme demonstrates its potential for different clinical contexts.

Due to COVID-19 pandemic eHCS training within ImpENSA Training Programme was delivered and evaluated exclusively online. On a global level, telemedicine in general and virtual nutrition counselling are becoming more common [28]. Through remote delivery of eHCS training on the Zoom platform, we were able to model desired behaviours in the online environment that likely resembles the current or future workplace of HCPs.

Theory-based evaluation follows a structured, systematic approach to investigate the effects and impact of a training programme. Applying Kirkpatrick’s evaluation model [18] allowed us to assess the effectiveness of eHCS training in supporting participants to achieve the intended outcomes, understand how study participants experienced the training and explore training features that facilitated learning (mindset and clinical practice changes towards person-centred care). Another important strength of this study is its mixed method design. By integrating both quantitative and qualitative methods, we obtained rich data allowing in-depth and objective exploration of our research questions and, subsequently, resulting in more comprehensive findings. Furthermore, utilising the established HCS evaluation tools that have been used for many studies globally allowed us to interpret our results in the context of previous HCS evaluation studies while additional questions enabling to explore ImpENSA Training Programme specific aspects.

Limitations include the modest study sample size and enrolment of mainly dietitians as study participants, who may not be representative of other healthcare professionals. Further, selection of participants through ADSA might have affected the representativeness of our sample, potentially not reaching the South African nutrition work force beyond this network. Finally, the results related to the application of HCS and changes in practice were self-reported by the participants.

After the pilot phase, the three South Africa HCS coordinators, together with selected educators/trainers from academic and healthcare institutions, were trained to additionally deliver in-person training for scaleup. The ImpENSA Training Programme is being implemented by the Centre of Excellence for Nutrition at North-West University, South Africa. The programme, hosted in the African Academy of Nutrition and Health training platform at LMU Munich (https://aanh.med.lmu.de/), is currently available for undergraduate and in-service HCPs. Within the programme, eHCS training is available upon registration and after successful completion of the eLearning modules.

## Conclusions

Better utilisation of HCPs’ potential for supporting healthy behaviours among their patients, in particular those from disadvantaged communities, is needed for improved health and more effective prevention and management of disease. eHCS training, integrated with knowledge-based eLearning modules presents a promising online training intervention to effectively enhance the way HCPs support behaviour change in their patients to ultimately optimise early life nutrition. The design approach applied to the ImpENSA Training Programme can be utilised in the development of training programmes contextualised for different topics to build competency in person-centred care and delivery of HCPs.

## Supporting information

S1 Fig ImpENSA training programme and module outlines. (PDF)

S1 Appendix eHCS module descriptor. (PDF)

S2 Appendix Evaluation tools–questionnaires and assessment. (PDF)

S3 Appendix Follow-up interview guide. (PDF)

S1 Checklist Inclusivity in global research. (DOCX)

S1 Table Sample size available for data analysis at baseline, pre-, post-eHCS training and at follow-up among those who completed the training (n = 27). (PDF)

S2 Table Application of ODQs. (PDF)

S1 Dataset Assessment and questionnaire dataset. (XLSX)
