# Correction: ImpENSA eHealthy Conversation Skills training for healthcare professionals aimed at improving micronutrient status during the first 1000 days in South Africa

**DOI:** 10.1371/journal.pgph.0005780

**Published:** 2026-01-05

**Authors:** Bernadeta Patro-Golab, Sunhea Choi, Jan Lukasik, Corinna Walsh, Maciej Kolodziej, Lize Havemann-Nel, Estelle Venter, Kerry Sexton, Selma Omer, Liz Goddard, Keith M. Godfrey, Wendy Lawrence, Berthold Koletzko, ImpENSA Study Group

The following ImpENSA Study Group members are missing from the Acknowledgement statement: Ali Dhansay, Corinna Walsh, Elize Symington, Christine Taljaard-Krugell.
