# Supplementary figures and images for: ImpENSA eHealthy Conversation Skills training for healthcare professionals aimed at improving micronutrient status during the first 1000 days in South Africa
Source: PLOS Glob Public Health. 2024 Dec 4;4(12):e0003833. doi: 10.1371/journal.pgph.0003833 (PMC11616819; doi:10.1371/journal.pgph.0003833)

**S1 Fig.** ImpENSA Training Programme and module outlines

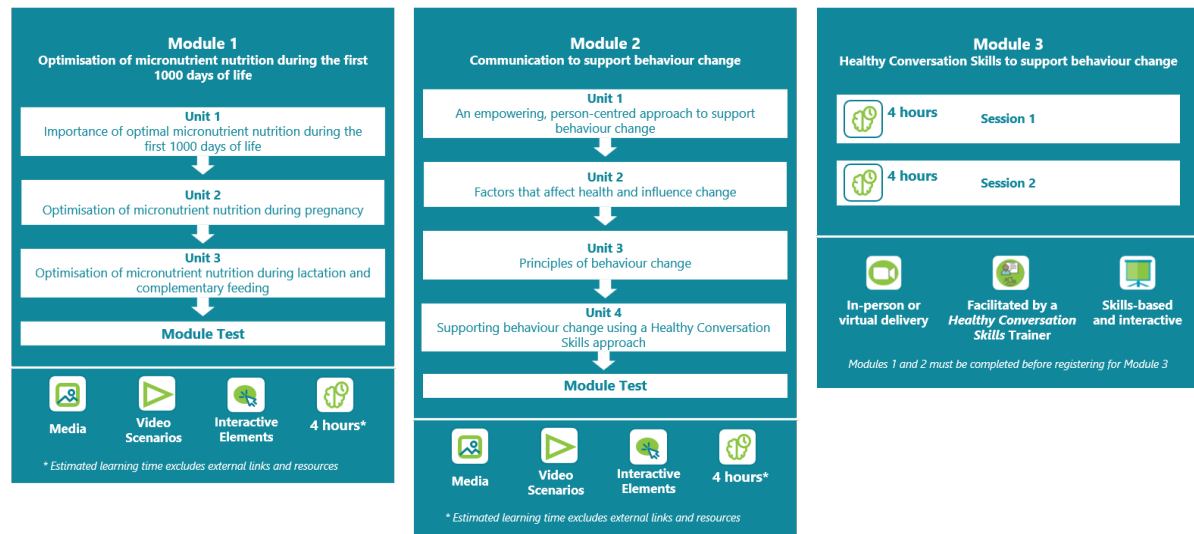

Supplement: S1 Fig — (PDF) [file pgph.0003833.s001.pdf]
