# Supplementary material for: ImpENSA eHealthy Conversation Skills training for healthcare professionals aimed at improving micronutrient status during the first 1000 days in South Africa
Source: PLOS Glob Public Health. 2024 Dec 4;4(12):e0003833. doi: 10.1371/journal.pgph.0003833 (PMC11616819; doi:10.1371/journal.pgph.0003833)
Supplement: S1 Appendix — (PDF) [file pgph.0003833.s002.pdf]

S1 Appendix. eHCS Module descriptor

ImpENSA Module 3: eHealthy Conversation Skills to support behaviour change

Module Profile

Module title	Healthy Conversation Skills (HCS) to support behaviour change (online)
NQF level(s)	**6**
Target groups	**Health Care Professionals (HCPs)** (i.e. dietitians/nutritionists, registered nurses/midwives, medical practitioners, pharmacists)
Mode of delivery	Online group training
Prerequisites	Fluency in English

**Learning time**	
Online facilitated training	2 x 3½ hours (7 hours)
Independent study	1 hour (for action-planning, practice & critical reflection between sessions)
Total hours	8 hours

**Pre-requisite, co-requisite and follow-up modules**	
Pre-requisite module(s):	• Module 1: Optimising Micronutrient Nutrition during the First 1000 Days of Life (eLearning)<br>• Module 2: Communication Skills to Support Behaviour Change (eLearning)
Co-requisite module(s):	None
Follow-up module(s):	Train-the-Trainers (Coordinators) for those who would like to become Coordinators of the ImpENSA programme

Module aims, learning outcomes and assessment criteria

Aims

The goal of the ImpENSA programme is to equip targeted HCPs with the necessary knowledge and skills to better support pregnant women, mothers and caregivers to optimise intake of key micronutrients during the first 1000 days of life. After HCPs have undertaken eLearning Module 1 (Optimising Micronutrient Nutrition during the First 1000 Days of Life) and eLearning Module 2 (Communication Skills to Support Behaviour Change) which provide the underpinning knowledge on micronutrient nutrition and behaviour change, **this online training aims** to introduce HCPs to a set of skills to increase their confidence and competence in supporting individuals to change their health behaviours in order to improve their health and well-being.

Learning outcomes and assessment criteria

**Module learning outcomes**	**Assessment criteria**
After completion of this module, the learner will be able to:<br><br>• Effect behaviour change using HCS to support pregnant women, parents and care-givers to identify and take first steps towards better health and well-being for themselves and their infants.<br>• Explore their own current practice and reflect on changes in their own behaviour over time in order to support change in others.<br>• Prioritise optimisation of micronutrient nutrition during the first 1000 days of life in your daily practice.	The learner proves that the outcomes are achieved if he/she:<br><br>• Uses HCS and Behaviour Change Techniques (BCTs) in his/her practice to support his/her clients/patients to change behaviour.<br><br>• Explores his/her own practices and plans changes<br>• Implements the planned changes.<br><br>• Prioritises support for behaviour change related to micronutrient nutrition during the first 1000 days of life in his/her daily practice.

Summary of syllabus content

- Introduction to four HCS competencies: asking Open Discovery Questions (ODQs), reflecting, listening and supporting SMARTER planning for change
- HCS philosophy
- Asking ODQs
- Reflecting on the learner’s own practice
- SMARTER planning for change
- Behaviour Change Techniques (BCTs)
- Feedback on use of HCS
- Structured approach to reflection using Gibb’s critical reflection model
- Focus on micronutrient nutrition during the first 1000 days of life

Detailed module outcomes and session delivery plan

This online module will consist of 2 half-days (2 x 3½ hours) with time between sessions for trainees to reflect on and practise the skills. Group size is ideally between 10-14 learners and the training will be delivered by two trainers. The theme of the training is generally based around healthy lifestyle behaviours but will be tailored to optimising micronutrient nutrition during the first 1000 days. The skills acquired can be used to support behaviour change in any area of practice.

Session 1: Getting to know you, your beliefs & your world

This session will introduce learners to the HCS training philosophy and skills.

Syllabus

- The four HCS competencies
- Wider Determinants of Health and how they impact health behaviour
- The HCS philosophy
- Asking Open Discovery Questions (ODQs)
- Real Play demonstration
- Introduction to SMARTER planning

Learning outcomes

After completion of this session, the learner will be able to:

- Describe four HCS competencies to support behaviour change.
- Demonstrate an understanding of how health behaviours are affected by the wider determinants of health.
- Formulate and ask ODQs to help his/her client/patient explore an issue.
- Spend more time listening to his/her clients/patients than giving information or making suggestions.
- Make a SMARTER plan to set their own goal

Session plan

Times	Activities [Overview]	SESSION 1: 3½ hours<br>Getting to know you, your beliefs & your world
**20 mins**	Welcome, housekeeping & introductions<br><br>Annotation practice	Ensure pre-training evaluation has been completed<br>Introduce Zoom functions to be used throughout the training<br>Round robin/ice-breaker in pairs in breakout rooms (2mins), find out 2-3 things about each other & then introduce/share with group. Facilitator introductions.<br>Outline the 4 HCS/learning outcomes & plan for training session: clarify expectations.

**30 mins**	**Activity 1**<br>Wider Determinants of Health (WDoH) & exploratory discussion	WDoH – facilitate small groups to explore understanding of each "level" in turn (in breakout rooms).<br>Drawing on these discussions, explore trainees' role in supporting behaviour change. What do people say about the challenges to adopting healthier behaviours? [Capture quotes/ scenarios]
**15 mins**	**Activity 2:1**<br>Having a conversation	In pairs in breakout rooms; recorded on trainees' phones. Use an example of own behaviour would like to change (something real/small, just shared in the pair). 2 mins in each role. 'Talker' (patient/client) & 'Helper' (practitioner) … (&'observer' if odd nos). This captures "baseline" conversation style to reflect on in A2.2.
**35 mins**	**Activity 3**<br>Beliefs axis	"Agree" & "Disagree" axis on slides (trainees use annotate function). 4 statements to explore<br>Trainees put their name on the axis depending on how strong their belief is. Facilitator explores these positions: if you hold this belief OR if you don't hold this belief, 'How would you support someone to change? What kind of practitioner does that make you?' Encourage group discussions.<br>What's the rationale for exploring these statements? Ensure fully explored before presenting the HCS philosophy – training may feel different: interactive, modelling the skills.
**10 mins**	**BREAK**	
**30 mins**	**Activity 4**<br>Developing exploratory questions	Quotes on slide prepared from A1; ask trainees to review & select one to work on. What would be your response to this statement? Each trainee annotates the slide with their response ...compare and contrast, with following Qs for group:<br>What kind of response is this? Question or statement? What kind? How might someone respond to this? Where does the conversation go? Talk through each category.<br>Repeat with another quote – prompt practise in writing an ODQ.<br>Present Response Styles Framework & explore with group the various types of responses & where they take you.
**10 mins**	**Activity 5**<br>Real Play	Ask for a volunteer with something real they'd like to change (perhaps what spoke about in pair earlier). Facilitator models 2 styles of conversation of 2 mins each: 1 = telling/suggesting/my own experiences; 2 = ODQs & listening. Volunteer, then group, reflect on differences & how this would look in real world practice.
**10 mins**	**Activity 2:2**<br>Listening back	Ask, "what would be good thing to do now?"<br>In same pairs in breakout rooms listen back to earlier recordings. Refer to Response Styles Framework, 'what strategies were you using? Any ODQs?' Discuss with group – future changes?
**15 mins**	**Activity 6**<br>Setting SMARTER goals	Introducing SMARTER planning –get group to develop a SMARTER goal for a particular health behaviour (maybe something that's come up already in group/pair discussions). Encouraging use of ODQs. Write around poster to illustrate process.
**25 mins**	**Activity 7**<br>Supporting SMARTER goal-setting	Provide link to SMARTER planning for change sheets.<br>Between 1 & 10, how important is it to practise using HCS (ODQs, listening, reflecting, setting SMARTER goals) before the next session? How/ when can you do this? In pairs in breakout rooms develop a SMARTER plan by asking each other ODQs. Will review sheets & any changes at start of next session. Can set HCS practice goal &/or personal behaviour change goal.

**10 mins**	Personal Reflection<br><br>THANKS!	Trainees reflect on the session. What is one aspect of today's training you've found useful? Round robin – 30secs each; can annotate slide<br><br>Ensure have any links including SMARTER planning for change sheet before Session 2.
**3½ hrs**	**End of Session 1**	

Session 2: Practising "Healthy Conversation Skills" & learning complementary strategies to support behaviour change

This session will consolidate learning from Session 1 and introduce learners to additional strategies to support behaviour change, and provide further opportunities to practise, feedback and reflect.

Syllabus

- Review of four HCS, philosophy of HCS, SMARTER planning
- Using ODQs to review a SMARTER plan for change
- Behaviour Change Techniques (BCTs)
- Introduction to feedback model
- Introduction to Gibbs' critical reflection model
- Supporting someone to change behaviour using HCS
- Planning the implementation of HCS in the learner's own practice

Learning outcomes

After completion of this session, the learner will be able to:

- Ask ODQs to support his/her clients/patients to make a SMARTER plan.
- Use a SMARTER plan for goal-setting and planning change with his/her clients/patients.
- Use BCTs to support behaviour change.
- Reflect and feedback on his/her own and others' practice and conversations.

Session plan

Times	Activities [Overview]	SESSION 2: 3½ hours<br>[Practising "Healthy Conversation Skills" & learning complementary strategies to support behaviour change]
**15 mins**	Welcome & Session 1 review	Reminder of technology.<br><br>Review session 1: What are the 4 HCS, Philosophy, SMARTER, Response Styles Framework – as group identify all elements of each.
**10 mins**	Beliefs Axis revisited	Repeat 'philosophy' axis – any movement? Explore 2 statements with group re any changes in beliefs, 'what prompted the group/individual to change position?'
**30 mins**	**Activity 8**<br><br>Reviewing SMARTER goal-setting	In pairs in breakout rooms review SMARTER planning for change sheets and any goals set (10min each, then swap over).<br><br>Practising ODQs to encourage each other to reflect on progress, challenges, successes etc (using 'Sharing stories about change' prompt). Highlight how this activity uses all HCS.<br><br>Facilitate group to share experiences & learning about the process of change.

**40 mins**	**Activity 9**<br><br>Behaviour Change Techniques	Facilitate group to share BCTs.<br><br>On blank slide note BCTs elicited from group – use prompts to draw these out.<br><br>Group to reflect on the list generated on slide. What strategies have you used to support someone to make a change? What have you tried personally? What has worked?<br><br>Final discussion in group on how to integrate BCTs with HCS.
**10 mins**	**BREAK**	
**20 mins**	**Activity 10**<br><br>SMARTER Team Challenge!	Exploring ODQs that support goal-setting & planning change. In 2 groups - 5 mins to ask facilitator ODQs to support them to make a SMARTER plan (incorporating BCTs).<br><br>One trainer to to note on slide, questions asked for each letter of SMARTER as it's addressed.<br><br>Other group observes. Swap over.<br><br>Reflect on both groups' performances. How hard was it to achieve a SMARTER plan? What worked well? How might you use this learning? What other questions could have been asked?
**35 mins**	**Activity 11**<br><br>Using HCS for effective feedback	Introduce model of feedback using HCS to avoid confrontation or defensiveness.<br><br>In 3s in breakout rooms, practise supporting someone to change their behaviour using HCS.<br><br>Using a scenario, 2 learners take the roles of practitioner & client (actors) whilst the third is observer/ timekeeper (3 mins). The role plays are followed by feedback for the "practitioner" (using HCS, eg "How did you think that went? What went well/was challenging? What could you have done differently?) provided by the observer and then the "client" (3 mins). Learners alternate roles with two more scenarios.<br><br>Group discussion to share learning and explore challenges/ tips.
**25 mins**	**Activity 12**<br><br>Practising critical reflection	Introduce Gibbs' critical reflection model.<br><br>In pairs in breakout rooms, take turns to go through the model anchoring reflection on a recent conversation that elicited a strong emotion (5 mins each).<br><br>Group discussion to explore any differences that might come from using a structured approach for reflection (eg getting stuck in the description or being blinded by feelings prevents analysing & learning from experiences).
**25 mins**	**Activity 13**<br><br>If-Then Plans<br><br>Personal reflection	Introduce If-Then Plans as way of making next steps concrete. In pairs in breakout rooms, take turns to set personal If-Then Plan (5 mins each)<br><br>Trainees annotate slide with reflections & feedback on the training. How will you use HCS in your own practice & what are your next steps? What have you gained from the training?
**10 mins**	Evaluation & final thoughts<br><br>THANK YOU	Remind to complete online post-training survey & course feedback sheets.<br><br>Final Q&A.
**3½ hrs**	**End of Session 2**	

Summary of assessment methods

Use of ODQs will be assessed by pre- and post-training evaluation sheets (consisting of assessment and questionnaire) completed by the trainees. Competence in the other three HCS can be assessed by using previously developed and published tools. This can be done using any of these methods:

- Standardised telephone/Zoom call to each trainee at a pre-determined time post-training, using standardised script and coding rubric.
- Observation of trainee with a client (could be done virtually if there are no F2F consultations) , using standardised observation tool and coding rubric .
- Reflective feedback interview with trainee following observed session with client, using standardised tool and coding rubric.
