# Supplementary material for: ImpENSA eHealthy Conversation Skills training for healthcare professionals aimed at improving micronutrient status during the first 1000 days in South Africa
Source: PLOS Glob Public Health. 2024 Dec 4;4(12):e0003833. doi: 10.1371/journal.pgph.0003833 (PMC11616819; doi:10.1371/journal.pgph.0003833)
Supplement: S2 Appendix — (PDF) [file pgph.0003833.s003.pdf]

S2 Appendix. Baseline, pre- and post-eHCS training evaluation tools – assessment and questionnaires (*adopted from the HCS training evaluation tools*)

Assessment (baseline, pre- and post-eHCS training evaluation), and baseline, post-eHCS training and follow-up questionnaires

Item 1. eHCS assessment at Baseline, Pre- and Post-eHCS training (*contextually relevant quotes were prepared for the present study)*

Below are four things individuals might say. Please write below the next thing you might say to support this individual to make a health behaviour change.

1. "I know that since I'm pregnant, I should eat "good foods", but it's hard for me to change my diet"

You say:

____

____

____

2. "The nurse tells me I should take prescribed ART (antiretroviral therapy) while I am breastfeeding, but I often forget to take it."

You say:

____

____

____

3. "My baby girl is already 4-month-old and I have to get back to work. So I need to stop breastfeeding."

You say:

____

____

____

4. "My mum tells me that breastmilk is not enough for my 3-month-old boy anymore. So, I am planning to give him some other foods from now."

You say:

____

____

____

Item 2. Baseline questionnaire (*the texts contextualised for the present study are in brackets.)*

On a scale of 1-10 please indicate how you feel in response to each question below by circling one number.

1. How confident do you feel about supporting **{** pregnant women, mothers/caregivers of young children **}** to make a change **{** in relation to micronutrient nutrition **}**?
   (Not confident) 1 2 3 4 5 6 7 8 9 10 (Very confident)

2. How important is it for you to support **{** pregnant women, mothers/caregivers of young children **}** to make a change **{** in relation to micronutrient nutrition **}**?
   (Not important) 1 2 3 4 5 6 7 8 9 10 (Very important)

3. How useful do you think the **{** consultations **}** you have are at supporting { pregnant women, mothers/caregivers of young children **}** to make a change **{** in relation to micronutrient nutrition **}**?
   (Not useful) 1 2 3 4 5 6 7 8 9 10 (Very useful)

Item 3. Post-questionnaire (*the texts contextualised for the present study are in brackets.)*

On a scale of 1-10 please indicate how you feel in response to each question below by circling one number.

1. How confident do you feel about using the skills you learnt on the HCS Training, in **{** consultations **}** with **{** pregnant women, mothers/caregivers of young children **}** to support them to make a change **{** in relation to micronutrient nutrition **}**?
   (Not confident) 1 2 3 4 5 6 7 8 9 10 (Very confident)

2. How important is it for you to support **{** pregnant women, mothers/caregivers of young children **}** to make a change **{** in relation to micronutrient nutrition **}**?
   (Not important) 1 2 3 4 5 6 7 8 9 10 (Very important)

3. How useful do you think the **{** consultations **}** you had are at supporting **{** pregnant women, mothers/caregivers of young children **}** to make a change **{** in relation to micronutrient nutrition **}**?
   (Not useful) 1 2 3 4 5 6 7 8 9 10 (Very useful)

4. How useful do you think the skills you learnt on the HCS Training will be for supporting **{** pregnant women, mothers/caregivers of young children **}** to make a change **{** in relation to micronutrient nutrition**}**?
   (Not useful) 1 2 3 4 5 6 7 8 9 10 (Very useful)

Feedback on training (*from the HCS training evaluation tools*)

Please provide your overall score for your HCS training experience (one being the lowest and five the highest):

1	2	3	4	5

1. What did you find particularly effective/enjoyable about the training?

2. How might the training be improved?

3. Any other comments on the training?

eHCS Trainer Evaluation (*from the HCS training evaluation tools*)

1. How well did the Trainers demonstrate the following?

(Please circle the number below - one being the lowest and five the highest score)

Trainer Name	Demonstrated Knowledge & Expertise					Reflection & Active Listening					Asked Open Discovery Questions					Made Content Relevant to me				
Trainer One	**1**	**2**	**3**	**4**	**5**	**1**	**2**	**3**	**4**	**5**	**1**	**2**	**3**	**4**	**5**	**1**	**2**	**3**	**4**	**5**
Trainer Two	**1**	**2**	**3**	**4**	**5**	**1**	**2**	**3**	**4**	**5**	**1**	**2**	**3**	**4**	**5**	**1**	**2**	**3**	**4**	**5**

2. What did you find particularly effective/helpful about your Trainers?

3. How might your Trainers improve?

4. Any other comments to the Trainers?

Item 4. Follow-up questionnaire (*the texts contextualised for the present study are in brackets.)*

On a scale of 1-10 please indicate how you feel in response to each question below by circling one number.

1. How confident do you feel about supporting **{** pregnant women, mothers/caregivers of young children **}** to make a change **{** in relation to micronutrient nutrition **}**?
   (Not confident) 1 2 3 4 5 6 7 8 9 10 (Very confident)

2. How confident do you feel about using Healthy Conversation Skills (HCS) in **{** consultations **}** with **{** pregnant women, mothers/caregivers of young children **}** to support them to make a change **{** in relation to micronutrient nutrition **}**?
   (Not confident) 1 2 3 4 5 6 7 8 9 10 (Very confident)

3. How important is it for you to support **{** pregnant women, mothers/caregivers of young children **}** to make a change **{** in relation to micronutrient nutrition **}**?
   (Not important) 1 2 3 4 5 6 7 8 9 10 (Very important)

4. How useful do you think the **{** consultations } you had since **{** ImpENSA Training Programme **}** were at supporting **{** pregnant women, mothers/caregivers of young children **}** to make a change **{** in relation to micronutrient nutrition **}?**
   (Not useful) 1 2 3 4 5 6 7 8 9 10 (Very useful)

5. How useful were the HCS when supporting **{** pregnant women, mothers/caregivers of young children **}** to make **{** a micronutrient nutrition behavior change **}**?
   (Not useful) 1 2 3 4 5 6 7 8 9 10 (Very useful)

Application of HCS in practice (*questions additionally developed for the present study*)

1. Please indicate whether you: 1 – strongly disagree, 2 – disagree, 3 – neither agree nor disagree, 4 – agree, 5 – strongly agree.

a. Since completing the ImpENSA Training Programme, I use Open Discovery Questions (‘what’ and ‘how’ questions) more often when supporting my patients to make a behaviour change

b. Since completing the ImpENSA Training Programme, I reflect on my practice and the way I consult my patients more often

c. Since completing the ImpENSA training, I listen to my patients more and spent less time than previously on giving information or making suggestions to my patients.

2. I use Open Discovery Questions ('what' and 'how' questions) in my practice (1 –never, 2 – rarely, 3 – sometimes, 4 – often, 5 – always):

a. To explore my patients' environments to identify barriers and facilitators for a behaviour change.
b. To help my patients identify solutions that are appropriate for their circumstances.
c. To support my patients in making a SMARTER plan for a behaviour change.
d. To support my patients in evaluating and reviewing their SMARTER plan for a behaviour change.

3. Please indicate (1 –never, 2 – rarely, 3 – sometimes, 4 – often, 5 – always):

a. Since completing the ImpENSA Training Programme, how often have you supported your patients to make a SMARTER plan for a behaviour change?

4. Since completing the ImpENSA Training Programme, I use SMARTER planning (yes/no):

a. I do not use SMARTER planning.
b. To prepare a behaviour change plan for my patients.
c. To support my patients to plan their behaviour change.
d. To help my patients to evaluate and review their behaviour change plans.
e. To plan and review my consultation to support the patients to change behaviour.
