# Supplementary material for: ImpENSA eHealthy Conversation Skills training for healthcare professionals aimed at improving micronutrient status during the first 1000 days in South Africa
Source: PLOS Glob Public Health. 2024 Dec 4;4(12):e0003833. doi: 10.1371/journal.pgph.0003833 (PMC11616819; doi:10.1371/journal.pgph.0003833)
Supplement: S3 Appendix — (PDF) [file pgph.0003833.s004.pdf]

S3 Appnedix. Follow-up interview guide

Follow-up interview guide (*relevant to eHCS training evaluation)*

Research questions:

1) How HCPs use HCS in their practice?
2) How useful HCS are in improving behaviour change support HCPs provide?

Interview participants: those who have completed the ImpENSA Training Programme

1. **Aims of the interview:**
   To explore
   a. Changes the participant made in terms of application of HCS in practice
   a. Use of four skills and competencies since the ImpENSA training
   b. HCS use and competencies before the training
2. Making the changes – reflective practice
   a. How the change was made/description of the process of change
3. How the changes worked for the patients – outcomes from changes made
   a. How application of HCS helped patients
   b. The context before the training
4. How the changes worked for them
   a. Benefits gained from the use of HCS
5. Participants' feedback on HCS training

Welcome: [Name of the participant] Thank you for your participation in the ImpENSA Training Programme follow-up study. You have completed the training few months ago. Today we'd like to hear how you have used what you gained from the ImpENSA Training Programme in your practice; what changes have you made in your practice and what has worked well for your patients and yourself; what were the barriers and challenges you have experienced in making these changes when supporting your patients in relation to micronutrient nutrition. We would also like to hear how useful you consider the ImpENSA Training Programme was for your practice.

Informed consent: As explained before (consent form; pre- and post-interview), we would like to record this interview. We will use the recording to compile a written version of the interview, or transcript. After the transcript, we will delete the recording. The research team will analyse the transcript together with other study data, for example questionnaire and assessment, to evaluate the ImpENSA Training Programme.

Do you agree that we can record the interview?

Guideline: During the interview, feel free to ask me questions. What you said during the interview will be kept confidential. Do you have any questions now? I will start recording now.

START RECORDING …

Opening question: What changes have you made in your practice since completing ImpENSA Training Programme?

{Questions about eLearning modules}

Key questions (and prompts as required)**:**

Before we continue, I just want to explain that by HCS we mean the following core skills:

- *Using open-ended ('open discovery') questions*
- *Reflecting on practice*
- *Active listening*
- *Supporting goal setting through SMARTER planning*

1. How have your consultations changed since attending the training in terms of HCS use?

   - What healthy conversation skills do you use in your practice?
   - How do you use the skills in your practice? (ask for an example)
     - i. How have you used ODQs in your consultations with pregnant women or mothers with babies?
     - ii. What HCS do you use to help your patients plan to make change and how do you use them?
     - iii. How have you helped them set SMARTER goals?
   - How has the proportion of time you spent on listening vs. giving information or making suggestions changed?
   - Have you reflected on your practice? If so, when and how you have done it? What were the outcomes?
   - How have you made the changes you have mentioned? Can you describe the process of change?
   - Of the changes you made what worked well?
   - How has the changes you made helped your patients?
   - How have they helped you?
   - What do you think you could do differently?

2. How useful was the HCS training in supporting you to change your practice?

   - What have you learnt from the HCS training?
   - What was most useful and what was not very useful?
   - How do you think you have used the skills you learnt during the HCS training?

{Questions about the ImpENSA Training Programme and overall study experience}

Ending question: Is there anything else you like to say or ask me before we finish?

Thank you!
