# Supplementary material for: ImpENSA eHealthy Conversation Skills training for healthcare professionals aimed at improving micronutrient status during the first 1000 days in South Africa
Source: PLOS Glob Public Health. 2024 Dec 4;4(12):e0003833. doi: 10.1371/journal.pgph.0003833 (PMC11616819; doi:10.1371/journal.pgph.0003833)
Supplement: S1 Checklist — (DOCX) [file pgph.0003833.s005.docx]

Inclusivity in global research

PLOS’ policy on inclusivity in global research aims to improve transparency in the reporting of research performed outside of researchers’ own country or community and ensures that PLOS publications reporting global research adhere to high standards for research ethics and authorship. Authors of relevant research articles may be asked to complete the questionnaire below, which outlines ethical, cultural, and scientific considerations specific to inclusivity in global research. This questionnaire may be requested when researchers have travelled to a different country to conduct research, if research uses samples collected in another country, research with Indigenous populations or their lands, or if research is on cultural artefacts. Researchers travelling to another country solely to use laboratory equipment will not normally be required to complete the questionnaire. However, the questionnaire can be requested at the journal’s discretion for any submission – if you have been requested to complete this questionnaire by the PLOS journal you submitted to, please do so.

Please complete the questionnaire below and include this as a Supporting Information file with your manuscript. Note that if your paper is accepted for publication, this checklist will be published with your article in the supporting information files. Please ensure that you reference the checklist in the main body of your manuscript. We suggest adding a subsection ‘Inclusivity in global research’ to your Methods section and adding the following sentence: “Additional information regarding the ethical, cultural, and scientific considerations specific to inclusivity in global research is included in the Supporting Information (SX Checklist)”

The questions have been designed to be applicable to a wide range of study types, and there are subsections for both human subjects research and non-human subjects research. If any of the questions are not relevant to your research please mark them as “N/A” as appropriate.

**Ethical considerations, permits and authorship**

*This section is applicable to all research types.*

Provide details as to who granted permissions and/or consent for the study to take place in the Methods section of your manuscript. This should include the names of **all** ethics boards, governmental organizations, community leaders or other bodies that provided approval for the study. If individuals provided approval refer to these people by their role or title but do not list their name(s).

| Reported on page number: 7 |
| --- |

If there were any deviations from the study protocol after approval was obtained, please provide details of these changes in the Methods section of your manuscript.

| Reported on page number: N/A |
| --- |

Did this study involve local collaborators that are residents of the country where the research was conducted or members of the community studied? If you do not have any authors from said communities, please provide an explanation for this below.

| Yes, the reported study involved local South African partners: North-West University, the University of Cape Town, Stellenbosch University, Association for Dietetics in South Africa and Nutrition Association of South Africa. |
| --- |

Everyone listed as an author should meet PLOS’ criteria for authorship and all individuals who meet these criteria should be included in the author byline, rather than the acknowledgements. For further information please see the journal’s Authorship Policy.

**Human subjects research (e.g. health research, medical research, cross-cultural psychology)**

Did you obtain written informed consent from a representative of the local community or region before the research took place? How did you establish who speaks for the community? Details of written informed consent obtained from study participants should be reported separately in the Methods section of your manuscript.

| The study was approved by the Human Research Ethics Committee of North-West University, South Africa.  The ImpENSA project was an EU Erasmus+ funded initiative and aimed to tackle the triple burden of malnutrition in Southern Africa through capacity building. The project consortium included five South African partners who effectively represented and spoke for the target community (South African healthcare and higher education sectors), and they were North-West University, Stellenbosch University, University of Cape Town, Association for Dietetics in South Africa, and Nutrition Society of South Africa). The Department of Health, South Africa and a small group of healthcare professionals and students had been engaged as part of key stakeholders (members of the project assembly) throughout the project. Additionally, healthcare and higher education institutions had been informed of the project and its progress and engaged at key stages of the ImpENSA Training Programme conceptualisation, development and implementation (integration of the training programme/modules into existing undergraduate health science training programmes and in-service/CPD training).  Activities in South Africa were led by North-West University in partnership with support from other South African and European partners, and these included the needs assessment for training, identification of training topics, target user engagement during the design and development of the ImpENSA training programme, review, testing and piloting of the training programme. Dissemination and sustainability were also led by South African partners with support from LMU.  Details of written informed consent obtained from study participants are reported in the Methods section of the manuscript (page 7). |
| --- |

How did members of the local community provide input on the aims of the research investigation, its methodology, and its anticipated outcome(s)?

| See above. |
| --- |

When engaging with the local community, how did you ensure that the informed consent documents and other materials could be understood by local stakeholders?

| The ImpENSA Training Programme pilot study was prepared and conducted collaboratively between European and South African partners. Medical University of Warsaw (evaluation lead), Poland; the University of Southampton, UK (co-evaluation); North-West University (South African project lead, pilot study coordination, training delivery and evaluation), Stellenbosch University (evaluation) and the Nutrition Society of South Africa (evaluation). All partners provided support, which included the appraisal and review of methodologies and evaluation tools, recruitment, etc.  The training programme was developed in English and fluency in English was one of the eligibility criteria for study participation. Therefore, we did not anticipate language related issues for the informed consent documents prepared in English. LHN and EV, North-West University communicated with healthcare professionals who expressed interest in the study and sent the informed consent documents. They responded to other questions/queries. |
| --- |

Will the findings of the research be made available in an understandable format to stakeholders in the community where the study was conducted (e.g. via a presentation, summary report, copies of publications, etc.)? Please provide details of how this will be achieved.

| Stakeholder engagement and dissemination have been key aspects of the ImpENSA project's sustainability strategies.  The training programme is currently hosted in the African Academy of Nutrition and Health training platform at LMU Munich (<https://aanh.med.lmu.de/>) and provides project updates as well as information about the training programme. Moreover, project activities and outputs/findings from the project have been communicated to stakeholders via project websites at LMU and partner institutions, biannual newsletters, social media (twitter, YouTube, etc.), presentations at conferences and currently journal publications. These communication activities will continue as part of ongoing sustainability strategies to scale up the implementation of the training programme in South Africa. |
| --- |

**Non-human subjects research using specimens/ animals collected as part of the study, or those housed in archival collections. Examples include archaeology, paleontology, botany and zoology.**

Did the permission you obtained from a local authority to perform the study include an agreement on access to outputs and benefit sharing? This may include procedures to enable fair distribution of the benefits and resources arising from the research performed. Please include any details of Prior Informed Consent and Benefit Sharing Agreements obtained. These may be required by field-specific regulations, for example the Convention on Biological Diversity (CBD) and the associated Nagoya Protocol.

| N/A |
| --- |

If the material used in your study was imported, please A) provide the year it was imported and B) indicate whether permits were obtained to import/export the materials used, C) provide details of any permits obtained. If this information is not available, please indicate this.

| N/A |
| --- |

If you used archival specimens, please state how the material used in your study was acquired by the institute it is held in and provide details of any permits obtained for the original excavations/ sample collection. If this information is not available, please indicate this.

| N/A |
| --- |

How was the potential cultural significance of the materials collected in your study to local communities considered in your research design? Were Indigenous peoples and/or local researchers and institutions involved with archaeological excavations / collection of specimens? If so, please provide a description of their involvement.

| N/A |
| --- |

If your manuscript includes photographs of human remains, please indicate whether authors obtained permission from descendants or affiliated cultural communities to do so.

| N/A |
| --- |
