# Supplementary material for: ImpENSA eHealthy Conversation Skills training for healthcare professionals aimed at improving micronutrient status during the first 1000 days in South Africa
Source: PLOS Glob Public Health. 2024 Dec 4;4(12):e0003833. doi: 10.1371/journal.pgph.0003833 (PMC11616819; doi:10.1371/journal.pgph.0003833)
Supplement: S1 Table — (PDF) [file pgph.0003833.s006.pdf]

S1 Table. Sample size available for data analysis at baseline, pre-, post-eHCS training and at follow-up among those who completed the training (n=27).

Data collection method	Baseline N (%)	Pre-eHCS N (%)	Post-eHCS N (%)	Follow-up N (%)
Assessment	26 (96)*	26 (96)*	26 (96)*	NA
Questionnaire	27 (100)	NA	26 (96)*	24 (92)
Interview	NA	NA	NA	24 (92)

NA, not applicable

*one participant out of 27 who completed eHCS training not included in the analysis due to technical issues with the assessment and questionnaire online data entry
