# Supplementary material for: ImpENSA eHealthy Conversation Skills training for healthcare professionals aimed at improving micronutrient status during the first 1000 days in South Africa
Source: PLOS Glob Public Health. 2024 Dec 4;4(12):e0003833. doi: 10.1371/journal.pgph.0003833 (PMC11616819; doi:10.1371/journal.pgph.0003833)
Supplement: S2 Table — (PDF) [file pgph.0003833.s007.pdf]

S2 Table. Application of ODQs (*Follow-up study, N=24*)

I use Open Discovery Questions (‘what’ and ‘how’ questions) in my practice (1 –never, 2 – rarely, 3 – sometimes, 4 – often, 5 – always):

	Mean	Never	Rarely	Sometimes	Often	Always
**a. To explore my patients’ environments to identify barriers and facilitators for a behaviour change.**	4.5	0 (0%)	0 (0%)	1 (4%)	10 (42%)	13 (54%)
**b. To help my patients identify solutions that are appropriate for their circumstances.**	4.6	0 (0%)	0 (0%)	1 (4%)	8 (33%)	15 (63%)
**c. To support my patients in making a SMARTER plan for a behaviour change.**	4.2	0 (0%)	1 (4%)	5 (21%)	9 (38%)	9 (38%)
**d. To support my patients in evaluating and reviewing their SMARTER plan for a behaviour change.**	4.2	0 (0%)	1 (4%)	5 (21%)	9 (38%)	9 (38%)
